# Supplementary material for: Detection of Cell-Fusing Agent virus across ecologically diverse populations of Aedes aegypti on the Caribbean island of Saint Lucia
Source: Wellcome Open Res. 2020 Dec 16;5:149. Originally published 2020 Jun 25. [Version 2] doi: 10.12688/wellcomeopenres.16030.2 (PMC8030115; doi:10.12688/wellcomeopenres.16030.2)
Supplement: Peer review history

# Peer review: Reviewer response for version 2

**DOI:** 10.21956/wellcomeopenres.18175.r43154

**Published:** 2021-04-07

**Authors:** Lícia Natal Fernandes

**Affiliations:** 1 Institute of Tropical Medicine, University of São Paulo, São Paulo, Brazil

The article is about an entomologycal survey conducted in Sant Lucia, a Caribbean island. Association between some environmental parameters and the counts of female *Aedes aegypti* mosquitoes was performed and a screening of arbovirus was also carried out. Higher counts of *Aedes aegypti* were significantly associated with elevation and mosquito counts in semi-urban settings were lower than urban settings. Nucleotide sequences of cell fusing agent virus, a insect specific flavivirus, was detected with a high prevalence in *Aedes agypti* pools. It was very important to perform such study considering the island where the study took place has the potential for occurrence of arbovirus outbreaks.

 This is a well written article and only minor changes are suggested, as follows.

 In the first paragraph authors say: “YFV is also transmitted by *Ae. aegypti* and can result in large urban outbreaks and rapid spread to distant locations”. In order to avoid confusion I would say something like: “YFV is also transmitted by *Ae. aegypti* in the urban circle of transmission, what can result in large urban outbreaks and rapid spread to distant locations where population has not been vaccinated yet”.

 In my opinion, citation of Figure 1 should be removed from the last paragraph of the introduction chapter because its title mentions the traps, which are only described in the methods. Therefore, the citation should be placed only in methods.

 It would have been more appropriate to use a taxonomic key to separate mosquitoes *Aedes* from other mosquitoes. However I believe results are correct as the authors confirmed the morphology results by molecular biology based on the ITS1 gene.

 In the Results, authors say “No *Aedes albopictus* females were collected”, what gives the impression that males might have been collected. So I think it would be more appropriate to say that no *Aedes albopictus* were collected.

 It’s not clear how many adults from collected larvae were analysed, if they were processed in pools or individually. Also, it would be very interesting to add information on the larvae collection (frequency of collection and kinds of breeding sites sampled – natural or artificial or both).

 In the last paragraph of the results, authors begin saying the number of mosquitoes analysed (381), but when they talk about the positive results for PCR, it seems that information is given in number of mosquito pools. It would be important to have the number of mosquito pools analysed in the text, together with the number of positive pools. Also, it should be informed that positivity is given in number of pools and not in number of individual mosquitoes, if that really is the case. As mosquitoes were processed in pools, it’s not possible to know the real number of positive mosquitoes, therefore authors should consider to calculate and inform the minimum infection rate.

 One limitation of the study is the fact that very small nucleotide sequences were analysed, nonetheless this is very well discussed by the authors, that even comment on the importance of sequencing the entire genome of the virus detected.

 Authors found significantly association between counts of *Aedes aegypti* and elevation although do not discuss it. If any explanation for that is available, it would be very important to be in the text.

 - Figure 1 – extended material says: “yellow pins represent semi-permanent traps”. Is it correct or would it be “permanent traps” instead of “semi-permanent traps”?

Is the work clearly and accurately presented and does it cite the current literature?

Yes

If applicable, is the statistical analysis and its interpretation appropriate?

I cannot comment. A qualified statistician is required.

Are all the source data underlying the results available to ensure full reproducibility?

Yes

Is the study design appropriate and is the work technically sound?

Yes

Are the conclusions drawn adequately supported by the results?

Yes

Are sufficient details of methods and analysis provided to allow replication by others?

Yes

Reviewer Expertise:

Entomology / Molecular biology applied to detection of protozooans and viruses in mosquitos.

I confirm that I have read this submission and believe that I have an appropriate level of expertise to confirm that it is of an acceptable scientific standard.

# Peer review: Reviewer response for version 2

**DOI:** 10.21956/wellcomeopenres.18175.r41856

**Published:** 2021-01-05

**Authors:** Jandouwe Villinger

**Affiliations:** 1 International Centre of Insect Physiology and Ecology (icipe), Nairobi, Kenya

The issues of the original version of the manuscript have now been sufficiently addressed.

Is the work clearly and accurately presented and does it cite the current literature?

Partly

If applicable, is the statistical analysis and its interpretation appropriate?

No

Are all the source data underlying the results available to ensure full reproducibility?

No

Is the study design appropriate and is the work technically sound?

Yes

Are the conclusions drawn adequately supported by the results?

No

Are sufficient details of methods and analysis provided to allow replication by others?

Yes

Reviewer Expertise:

Molecular entomology of mosquito- and tick-borne diseases.

I confirm that I have read this submission and believe that I have an appropriate level of expertise to confirm that it is of an acceptable scientific standard.

# Peer review: Reviewer response for version 1

**DOI:** 10.21956/wellcomeopenres.17585.r40686

**Published:** 2020-10-23

**Authors:** Marco Neira

**Affiliations:** 1 The Cyprus Institute, Nicosia, Cyprus

**Major Revisions:**

 By comparing Table 1 with the mosquito trapping data available on the supplementary files, it would seem that most locations sampled with temporary traps were only sampled for a single day, with a single trap each, while locations sampled with permanent traps had a much higher number of trapping nights. If this was the case, the authors should address whether this style of sampling is appropriate for performing the type of statistical analysis presented in the paper. In any case, I suggest they include in Table 1 the number of trapping nights for each location.

 The data shown in Figure 2 and Table 1 suggest that one of the permanent trapping sites (Soufriere Hospital) consistently produced much higher yields than any of the other sites, including those sampled with temporary traps. However, the authors don’t discuss potential explanations for this phenomenon, or the ways in which this fact could (or not) create bias on their data analysis. I suggest they revise the discussion addressing these observations.

 As the authors correctly point out, the detection of flavivirus-like RNA in a mosquito could very well be the product of a transcribed cell silent agents (CSA) integrated in the insect’s genome sometime in the past. While it is certainly possible that a novel, extant virus could be the source of such CSA, it is also possible that the sequences obtained by the authors represent a CSA originating from a known virus and subsequently mutated after insertion in the mosquito genome. Alternatively, the sequences could correspond to a CSA derived from an extinct virus. In any case, I think that in the absence of a full viral genome, the authors should be more careful about their claim of having identified a novel insect-specific virus.

 The ‘Conclusions’ section discusses aspects such as the spread of arboviral diseases, the limited options available for vector control, the history of Zika outbreaks (among other), which are at best tangentially related to the paper’s original results. I recommend that the authors re-write this section, presenting conclusions that are actually related to their original work.

**Minor Revisions:**

 Throughout the paper, please italicize scientific names.

 Introduction:

- The authors state that “22 countries and territories have now been identified to have autochthonous transmission”, which could be interpreted as being a global tally of countries reporting Zika transmission. However, this number (a) corresponds exclusively to the Americas, and (b) represents an outdated figure. The latest figures available from the Pan-American Health Organization (available here) indicate that just within the Americas, 48 countries and territories have reported mosquito-borne Zika transmission. Considering that earlier on the paper the authors provide global data for the epidemiology of dengue, I would recommend revising this figure to reflect updated global data on Zika distribution. One potential source is the World Health Organization’s Zika Epidemiology Update (available here).
- The authors state that “widespread insecticide resistance has developed in Ae. aegypti, including high pyrethroid resistance rates in South America and further north in the Caribbean”. To back up this statement, the authors cite a single paper focused on resistance found on a specific location in Brazil, and one paper focusing on a location in the Caribbean. I would recommend that the authors increase the number of citations to better represent the actual spread of pyrethroid resistance in the region. Suggestions include:
  Fonseca-Gonzalez *et al.* (2011) ^1^
  Rawlins SC (1998) ^2^ 
  Ryan SJ *et al*. (2019) ^3^

Methods:

- The authors should provide a clearer summary of their trapping strategy. In the current text, they state that trapping was performed using four permanent traps and four temporary traps, for what can be understood as a total of eight traps per each 24-hour period. However, mosquito trapping data available on the supplementary files suggests that on most dates only six or less traps were used.
- Please specify which taxonomic key was used to separate mosquito genera. If no specific key was used, please provide a complete list of characters used to sort specimens by genus.
- The legend in Figure 1 makes reference to an inset showing a “representative BG Sentinel 2 trap placed in the Des Cartier rainforest”. However, the actual figure does not contain any inset showing a representative trap.
- Please specify what kind of primers were used for reverse-transcription reaction.

Discussion:

-  In the first paragraph of the discussion, the sentence “The principle vector of these arboviral diseases…” should be changed to “The principal vector of these arboviral diseases…”.

Is the work clearly and accurately presented and does it cite the current literature?

Partly

If applicable, is the statistical analysis and its interpretation appropriate?

I cannot comment. A qualified statistician is required.

Are all the source data underlying the results available to ensure full reproducibility?

Yes

Is the study design appropriate and is the work technically sound?

Partly

Are the conclusions drawn adequately supported by the results?

No

Are sufficient details of methods and analysis provided to allow replication by others?

Partly

Reviewer Expertise:

Medical entomology, mosquito biology, mosquito control, vector/pathogen interaction, mosquito molecular physiology, insect transgenesis.

I confirm that I have read this submission and believe that I have an appropriate level of expertise to confirm that it is of an acceptable scientific standard, however I have significant reservations, as outlined above.

## References

[1] : Insecticide resistance status of Aedes aegypti (L.) from Colombia. *Pest Manag Sci*.2011;67(4) : 10.1002/ps.2081 430-7 10.1002/ps.2081 21394876

[2] : Spatial distribution of insecticide resistance in Caribbean populations of Aedes aegypti and its significance. *Rev Panam Salud Publica*.1998;4(4) : 10.1590/s1020-49891998001000004 243-51 10.1590/s1020-49891998001000004 9924507

[3] : Seasonal and geographic variation in insecticide resistance in Aedes aegypti in southern Ecuador. *PLoS Negl Trop Dis*.13(6) : 10.1371/journal.pntd.0007448 e0007448 10.1371/journal.pntd.0007448 31181073PMC6586360

# Response

**Published:** 2020-12-15

**Authors:** Thomas Walker

**Affiliations:** London School of Hygiene & Tropical Medicine, UK

Response to reviewer 2

**Many thanks Marco for your thorough and thoughtful review.  Our responses are shown below in bold.**

 Major Revisions:

 By comparing Table 1 with the mosquito trapping data available on the supplementary files, it would seem that most locations sampled with temporary traps were only sampled for a single day, with a single trap each, while locations sampled with permanent traps had a much higher number of trapping nights. If this was the case, the authors should address whether this style of sampling is appropriate for performing the type of statistical analysis presented in the paper. In any case, I suggest they include in Table 1 the number of trapping nights for each location.

**Yes this is correct and why we described some as temporary and others as permanent.  We repeated our statistical analysis using a negative binomial mixed effects model (as opposed to a** **generalized linear model)** **to account for** **the potential additional random effect of trap site (repeat sampling from the permanent traps but not the temporary traps) and the results remained relatively unchanged.  We found a statistically increased mosquito count for higher elevations and for urban (vs semi-urban) areas but no statistical association between counts and either humidity or temperature.  As these significant associations are the same, the results of this analysis are now presented in Supplementary Table 2, *extended data*).** **We have also now included the trapping nights for each location in Table 1.**

 The data shown in Figure 2 and Table 1 suggest that one of the permanent trapping sites (Soufriere Hospital) consistently produced much higher yields than any of the other sites, including those sampled with temporary traps. However, the authors don’t discuss potential explanations for this phenomenon, or the ways in which this fact could (or not) create bias on their data analysis. I suggest they revise the discussion addressing these observations.

**Soufriere Hospital did indeed produce higher yields and we have added three sentences at the start of the second paragraph in the discussion to show how the trapping location had several optimal characteristics for *Ae. aegypti.***

 As the authors correctly point out, the detection of flavivirus-like RNA in a mosquito could very well be the product of a transcribed cell silent agents (CSA) integrated in the insect’s genome sometime in the past. While it is certainly possible that a novel, extant virus could be the source of such CSA, it is also possible that the sequences obtained by the authors represent a CSA originating from a known virus and subsequently mutated after insertion in the mosquito genome. Alternatively, the sequences could correspond to a CSA derived from an extinct virus. In any case, I think that in the absence of a full viral genome, the authors should be more careful about their claim of having identified a novel insect-specific virus.

**We agree and have modified the end of our discussion to provide a more balanced view. Additional CFAV sequences which became publicly available after the original analysis have now enabled a re-analysis to be carried out which has been incorporated into the latest version of the manuscript, with relevant areas updated.**

 The ‘Conclusions’ section discusses aspects such as the spread of arboviral diseases, the limited options available for vector control, the history of Zika outbreaks (among other), which are at best tangentially related to the paper’s original results. I recommend that the authors re-write this section, presenting conclusions that are actually related to their original work.

**We agree and have modified our conclusions to include some sentences on insect-specific flaviviruses.  However, confirmation of a wide distribution of *Ae. aegypti* on the island of St Lucia as part of our entomology survey and screening for human arboviruses undertaken does (in our opinion) relate to the possibility for future arboviral outbreaks.**

**Minor Revisions:**

 Throughout the paper, please italicize scientific names.

**We have done this.**

 Introduction:

- The authors state that “22 countries and territories have now been identified to have autochthonous transmission”, which could be interpreted as being a global tally of countries reporting Zika transmission. However, this number (a) corresponds exclusively to the Americas, and (b) represents an outdated figure. The latest figures available from the Pan- American Health Organization (available here) indicate that just within the Americas, 48 countries and territories have reported mosquito-borne Zika transmission. Considering that earlier on the paper the authors provide global data for the epidemiology of dengue, I would recommend revising this figure to reflect updated global data on Zika distribution. One potential source is the World Health Organization’s Zika Epidemiology Update (available here).

**Many thanks for pointing this out and we have updated our figures based on the last available WHO update (July 2019). **

- The authors state that “widespread insecticide resistance has developed in Ae. aegypti, including high pyrethroid resistance rates in South America and further north in the Caribbean”. To back up this statement, the authors cite a single paper focused on resistance found on a specific location in Brazil, and one paper focusing on a location in the Caribbean. I would recommend that the authors increase the number of citations to better represent the actual spread of pyrethroid resistance in the region. Suggestions include: Fonseca-Gonzalez *et al. *(2011)1 , Rawlins SC (1998)2 , Ryan SJ *et al*. (2019)3

**Many thanks for these suggestions to better represent pyrethroid resistance and we have incorporated these references.**

 Methods:

- The authors should provide a clearer summary of their trapping strategy. In the current text, they state that trapping was performed using four permanent traps and four temporary traps, for what can be understood as a total of eight traps per each 24-hour period. However, mosquito trapping data available on the supplementary files suggests that on most dates only six or less traps were used.

**Yes that is correct and this was due to logistical difficulties** **accessing all sites across the island so not all trapping days had four temporary traps running synchronous with the four longer-term permanent traps.  Our methods have now been modified for clarity. **

- Please specify which taxonomic key was used to separate mosquito genera. If no specific key was used, please provide a complete list of characters used to sort specimens by genus.

**In our methods we have the following sentences:**

**‘Trapped mosquitoes were collected, killed on ice for genera morphological identification to identify individuals belonging to the Aedes genus using basic defining characteristics including patterns of light and dark scales on the abdomen and thorax and alternating light and dark bands on the legs** **.’**

**We then used** **ITS1 real-time PCR assays that discriminates between *Ae. aegypti* and *Ae. albopictus*.  **

- The legend in Figure 1 makes reference to an inset showing a “representative BG Sentinel 2 trap placed in the Des Cartier rainforest”. However, the actual figure does not contain any inset showing a representative trap.

**Thank you for pointing this out – this was error from a previous figure version and we have removed this reference to the trap from the figure legend. **

- Please specify what kind of primers were used for reverse-transcription reaction.

**We have added in the methods that random primers are used with the** **QuantiTect Reverse transcription Kit **

- Discussion: In the first paragraph of the discussion, the sentence “The principle vector of these arboviral diseases...” should be changed to “The principal vector of these arboviral diseases...”.

**Thank you for spotting this error and we have amended this**

# Peer review: Reviewer response for version 1

**DOI:** 10.21956/wellcomeopenres.17585.r39242

**Published:** 2020-07-14

**Authors:** Jandouwe Villinger

**Affiliations:** 1 International Centre of Insect Physiology and Ecology (icipe), Nairobi, Kenya

This is a generally well written manuscript on a study surveying mosquito viruses in St Lucia. Although no arboviruses were detected, they found a 19% prevalence of Aedes-specific flaviviruses. There is a general lack in the literature of information on individual prevalence of insect-specific flaviviruses in mosquito populations. Therefore, their infection rate data could be highlighted more in the paper

 My main concern is that the authors are likely over interpreting their finding of a new Aedes-specific flavivirus (AeFV) sequence as a novel insect-specific flavivirus. Just a few CFAV sequences and only one other AeFV sequence are included in the phylogenetic analyses. CFAV is part of a clade of AeFVs that are broadly distributed and show variation. The Saint Lucia AeFV seem to fall within the expected variation of AeFVs, if not even CFAVs. At the very least the sequence they found simply represents a new strain of AeFV associated with *Aedes aegypti* in St. Lucia. Percent nucleotide homologies are not given, so I cannot fully judge, but these are likely a St. Lucia lineage of CFAV. It would also be useful if there were GenBank accession numbers associated with the study’s ISF sequences so that their phylogenies can be independently assessed and the sequences can be used in future analyses of ISF variation/evolution.

 Please consider the recent papers:

 Patterson *et al*. (2020) ^1^.

 Baidaliuk *et al*. (2020) ^2^. 

 Minor comments:

- The authors should ensure that conventions are followed and that genus and species names are italicized (double check all ‘ *Ae. aegypti’* and ‘ *Alphavirus*’).
- It is not clear to me why Figure 3 is required as the same information is fully represented in Figure 4. I think that Figure 3 can be removed.

Is the work clearly and accurately presented and does it cite the current literature?

Partly

If applicable, is the statistical analysis and its interpretation appropriate?

No

Are all the source data underlying the results available to ensure full reproducibility?

No

Is the study design appropriate and is the work technically sound?

Yes

Are the conclusions drawn adequately supported by the results?

No

Are sufficient details of methods and analysis provided to allow replication by others?

Yes

Reviewer Expertise:

Molecular entomology of mosquito- and tick-borne diseases.

I confirm that I have read this submission and believe that I have an appropriate level of expertise to confirm that it is of an acceptable scientific standard, however I have significant reservations, as outlined above.

## References

[1] : Exploiting insect-specific viruses as a novel strategy to control vector-borne disease. *Curr Opin Insect Sci*.2020;39: 10.1016/j.cois.2020.02.005 50-56 10.1016/j.cois.2020.02.005 32278312PMC7302987

[2] : Novel genome sequences of cell-fusing agent virus allow comparison of virus phylogeny with the genetic structure of Aedes aegypti populations. *Virus Evol*.2020;6(1) : 10.1093/ve/veaa018 veaa018 10.1093/ve/veaa018 32368352PMC7189118

# Response

**Published:** 2020-12-15

**Authors:** Thomas Walker

**Affiliations:** London School of Hygiene & Tropical Medicine, UK

Response to reviewer 1

**Many thanks Jandouwe for your thoughtful review.  Our responses are shown below in bold:**

 My main concern is that the authors are likely over interpreting their finding of a new Aedes-specific flavivirus (AeFV) sequence as a novel insect-specific flavivirus. Just a few CFAV sequences and only one other AeFV sequence are included in the phylogenetic analyses. CFAV is part of a clade of AeFVs that are broadly distributed and show variation. The Saint Lucia AeFV seem to fall within the expected variation of AeFVs, if not even CFAVs. At the very least the sequence they found simply represents a new strain of AeFV associated with *Aedes aegypti* in St. Lucia. Percent nucleotide homologies are not given, so I cannot fully judge, but these are likely a St. Lucia lineage of CFAV. It would also be useful if there were GenBank accession numbers associated with the study’s ISF sequences so that their phylogenies can be independently assessed and the sequences can be used in future analyses of ISF variation/evolution.

 Please consider the recent papers: Patterson *et al*. (2020), Baidaliuk *et al*. (2020)

**We agree that although we included all relevant sequences that we were able to obtain at the time of original analysis, on which we based our original results and conclusions, there have since been further CFAV sequences made publicly available. We have therefore undertaken a reanalysis which now indicates our sequences are identical or very closely related to the CFAV sequence from Guadeloupe (From Baidaliuk et al. 2020)** **^1^** **and alongside this sequence may potentially form a Caribbean lineage of CFAV. We have adjusted our manuscript in all relevant areas to reflect this and replaced the previous Figure 3 and 4 phylogenetic trees with a single updated phylogenetic tree (now Figure 3) from the re-analysis which incorporates additional more recently available sequences of CFAV for a more comprehensive comparison.**

**We have now also included our % nucleotide homologies following re-analysis, in addition to further sequencing analysis information, within the Jeffries et al. qPCR and sequencing analysis file in the *Underlying data*.**

**As we sequenced products from a Pan-flavivirus PCR assay which detects a wide range of viruses** **^2^** **, we obtained sequences less than 200bp and therefore Genbank unfortunately will not accept these.  However, as stated in the underlying data section, raw sequence data is available at** **https://doi.org/10.17605/OSF.IO/DACKS** **.**

 Minor comments:

- The authors should ensure that conventions are followed and that genus and species names are italicized (double check all ‘ *Ae. aegypti’* and ‘ *Alphavirus*’).

**We have checked and corrected our manuscript for these conventions. **

- It is not clear to me why Figure 3 is required as the same information is fully represented in Figure 4. I think that Figure 3 can be removed

**We agree and as mentioned above we have removed both the previous Figure 3 and 4 and instead replaced them with an updated phylogenetic tree as a new Figure 3.**

**References**

 1          Baidaliuk, A. *et al.* Novel genome sequences of cell-fusing agent virus allow comparison of virus phylogeny with the genetic structure of Aedes aegypti populations. *Virus Evol* **6**, veaa018, doi:10.1093/ve/veaa018 (2020).

 2          Johnson, N. *et al.* Assessment of a novel real-time pan-flavivirus RT-polymerase chain reaction. *Vector Borne Zoonotic Dis* **10**, 665-671, doi:10.1089/vbz.2009.0210 (2010).
